# United States Veterinary Medical Association

**Published:** 1895-01

**Authors:** 


A National Charter for the United States Veterinary Medical Association.—The committee having in charge the securing of an act of incorporation for our National Association are laboring hard with the Senate Committee on Agriculture and Forestry, to which this bill was referred, and it behooves every member of the Association to assist the committee in every way possible. They have made a strong appeal for its concession on the grounds that it would aid the veterinarians and also the veterinary schools of America. Reference is made in their appeal to the fact that America is the only country that does not recognize the veterinarian, nor contribute anything for the support of schools, nor accord them any grade in the army beyond the rank of a private. Reference is made to the independent education of the American veterinarians and to their success in dealing with contagious and infectious diseases of the country, as well as their usefulness in assisting the exportation of cattle and meat abroad.

The non-political character of the bill has been referred to, and it is hoped that every member of the profession, and particularly those who are residents in the States where the members of said committee reside, will at least write them a letter urging consideration of this measure.

We herewith publish the names of said committee and the States of which they are residents. Senate Committee on Agriculture and Forestry: James Z. George, of Mississippi; Wm. B. Bate, of Tennessee; Matt. W. Ransom, of North Carolina; Wm. A. Peffer, of Kansas; Wm. N. Roach, of North Dakota; Jas. McMillan, of Michigan; Wm. D. Washburn, of Minnesota; Redfield Proctor, of Vermont; H. C. Hansbrough, of North Dakota.

